# Neuronal endolysosomal transport and lysosomal functionality in maintaining axonostasis

**DOI:** 10.1083/jcb.202111077

**Published:** 2022-02-10

**Authors:** Joseph C. Roney, Xiu-Tang Cheng, Zu-Hang Sheng

**Affiliations:** 1 Synaptic Function Section, The Porter Neuroscience Research Center, National Institute of Neurological Disorders and Stroke, National Institutes of Health, Bethesda, MD

## Abstract

Lysosomes serve as degradation hubs for the turnover of endocytic and autophagic cargos, which is essential for neuron function and survival. Deficits in lysosome function result in progressive neurodegeneration in most lysosomal storage disorders and contribute to the pathogenesis of aging-related neurodegenerative diseases. Given their size and highly polarized morphology, neurons face exceptional challenges in maintaining cellular homeostasis in regions far removed from the cell body where mature lysosomes are enriched. Neurons therefore require coordinated bidirectional intracellular transport to sustain efficient clearance capacity in distal axonal regions. Emerging lines of evidence have started to uncover mechanisms and signaling pathways regulating endolysosome transport and maturation to maintain axonal homeostasis, or “axonostasis,” that is relevant to a range of neurologic disorders. In this review, we discuss recent advances in how axonal endolysosomal trafficking, distribution, and lysosomal functionality support neuronal health and become disrupted in several neurodegenerative diseases.

## Teaser

Roney et al. review recent advances in how axonal endolysosomal trafficking, distribution, and functionality maintain distal degradation capacity in neuronal health and become disrupted in several neurodegenerative diseases.

## Introduction

Lysosomes are dynamic, membrane-bound, acidic organelles that play a central role in the degradation of intracellular and extracellular cargos. More than 60 hydrolytic enzymes that are active under acidic environments within the lysosomal lumen facilitate degradation of complex biological macromolecules, including proteins, lipids, nucleic acids, and carbohydrates (Perera and Zoncu, 2016; Settembre et al., 2013). Lysosomes are also composed of >200 integral membrane proteins important for many aspects of lysosome function, including acidification, ion and molecule transport, membrane contact and fusion, and intracellular transport (Ballabio and Bonifacino, 2020). These properties are essential for lysosome maturation, substrate catabolism, and lysosomal recycling critical to maintain cellular homeostasis (Luzio et al., 2007; Saftig and Klumperman, 2009). Defects in lysosome function are associated with rare and common neurodegenerative diseases, highlighting the importance of lysosomes to neuron health and survival (Ferguson, 2019; Nixon et al., 2008; Platt et al., 2018; Sharma et al., 2018).

Neurons are highly polarized cells consisting of a cell body, complex dendritic arbors, and a single long axon with extensive branches and terminals that can span several tens to hundreds of centimeters in length. Given this extended morphology, neurons require efficient bidirectional transport mechanisms to coordinate clearance of endocytic and autophagic cargos generated distally by lysosomes that are relatively enriched in the cell body. Efficient transport is critically important in maintaining neuron growth, survival, and function but particularly challenging in distal axons and terminal branching regions. Accumulation of autophagic vacuoles (AVs) and lysosome-like organelles characterizes axonal pathology in several neurodegenerative diseases associated with lysosome dysfunction, reflecting disruptions at various steps in the maturation and trafficking of endolysosomal and autophagic organelles (Gowrishankar et al., 2015; Lee et al., 2011; Roney et al., 2021; Tammineni et al., 2017b; Wong and Holzbaur, 2014; Xie et al., 2015). In this review, we provide an overview of axonal endolysosome maturation and trafficking, reconcile practical guidelines for labeling degradative lysosomes in nervous systems, discuss recent insights into how neurons have adapted the endolysosomal system to cope with their polarized morphology and maintain axonostasis, and summarize current knowledge about the mechanisms regulating axonal endolysosome transport and lysosomal distribution and functionality. We then discuss emerging lines of evidence implicating lysosome dysfunction and transport defects to the pathogenesis of neurodegenerative diseases across the age spectrum and provide perspective on whether recovering axonostasis would be an effective therapeutic approach. Additional insights and perspectives can be found in other in-depth reviews on endolysosomal trafficking (Ferguson, 2018; Ferguson, 2019; Lie and Nixon, 2019; Winckler et al., 2018; Winckler and Yap, 2011), axonal signaling endosomes (Barford et al., 2017; Yamashita, 2019; Yamashita and Kuruvilla, 2016), lysosome biogenesis and fusion/fission (Luzio et al., 2007; Saffi and Botelho, 2019; Saftig and Klumperman, 2009; Yang and Wang, 2021), lysosomal storage diseases (Marques and Saftig, 2019; Parenti et al., 2021; Platt et al., 2012; Platt et al., 2018), lysosome trafficking in nonneuronal cells (Bonifacino and Neefjes, 2017; Pu et al., 2016), and other emerging areas of lysosomal biology (Ballabio and Bonifacino, 2020; Lim and Zoncu, 2016; Saftig and Puertollano, 2021; Thelen and Zoncu, 2017).

## Endolysosomal and autolysosomal trafficking and maturation

**Figure 1.**
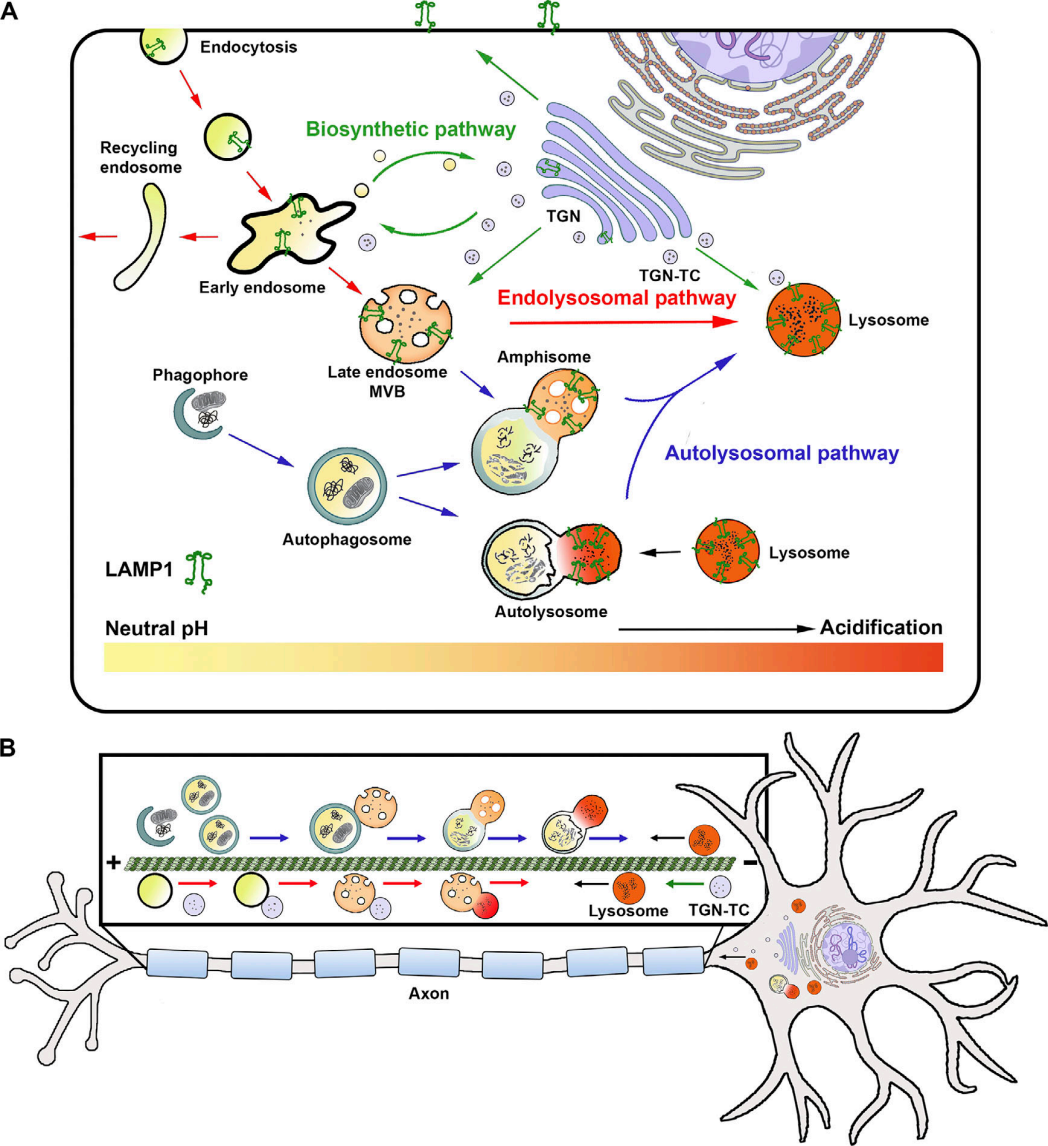
**Overview of endolysosomal and autolysosomal trafficking and maturation. (A)** Schematic summarizing membrane trafficking routes to the lysosome. Lysosomes receive cargos through the biosynthetic, endocytic, and autophagic pathways. Newly synthesized lysosomal proteins are delivered from the TGN to endosomes and lysosomes via TGN-derived TCs of the biosynthetic pathway (green arrows). Extracellular materials internalized by endocytosis that are destined for clearance reach lysosomes through the endocytic pathway; extracellular cargos not destined for lysosomes are sorted at the EE and recycled back to the plasma membrane (red arrows). Intracellular components targeted for degradation are sequestered within autophagosomes and delivered to lysosomes through the autophagic pathway (blue arrows). Note that newly synthesized lysosomal hydrolase precursors and membrane proteins may also be delivered to the plasma membrane, then internalized by endocytosis and subsequently trafficked to the lysosome through the endocytic pathway. Cross talk between these trafficking routes generates endocytic and autophagic intermediates that have varying luminal pH and LAMP1 content. These organelles mature into degradative compartments by passing through a continuum of intermediates that sequentially exchange membrane constituents and add lysosomal hydrolases. **(B)** Schematic showing bidirectional axonal transport that facilitates efficient maturation and clearance of autophagic and endocytic cargos. Top: Axonal autophagosomes are generated in axonal terminals and mature during their retrograde transport toward the soma through fusion events with LEs (blue arrows) or with degradative lysosomes delivered from the soma (black arrow). Bottom: Endocytic substrates transit through EEs and LEs as they move retrogradely toward the soma (red arrows), acquiring lysosomal components either from TGN-derived TCs (green arrow) or through fusion with lysosomes delivered from the soma (black arrow).

Lysosomes serve as terminal degradation hubs for endocytic and autophagic components. Extracellular materials internalized by endocytosis, intracellular components sequestered by autophagy, and newly synthesized lysosomal proteins reach endolysosomes through highly regulated trafficking routes. Endolysosomal trafficking from early endosomes (EEs) to late endosomes (LEs) and finally to mature lysosomes is essential for delivering target endosomal proteins for degradation (Fig. 1 A). Transition from EE to LE is marked by replacement of the small GTPase Rab5 with Rab7, as well as changes in membrane phosphoinositide composition (Gillooly et al., 2000; Liu et al., 2016; Poteryaev et al., 2010; Rink et al., 2005; Schink et al., 2016; van der Beek et al., 2022). In addition, several distinct mechanisms of the biosynthetic pathway sort and deliver newly synthesized lysosomal hydrolase precursors and integral membrane proteins from the TGN to endosomes and lysosomes (Geuze et al., 1985; Griffiths et al., 1988; Griffiths et al., 1990; Ishidoh and Kominami, 2002; Lobel et al., 1989). Dynamic cross talk between the endolysosomal and biosynthetic pathways enables lysosome biogenesis and maturation into degradative organelles. Moreover, intracellular materials sequestered within autophagosomes undergo stepwise maturation through fusion events with LEs or lysosomes to form degradative autolysosomes (Cheng et al., 2015; Klionsky and Emr, 2000; Levine and Klionsky, 2004; Maday et al., 2012). Thus, endocytic and autophagic organelles mature into degradative compartments by passing through a continuum of intermediates that sequentially exchange membrane constituents and add lysosomal hydrolases (Fig. 1 A). These heterogeneous intermediates, having diverse qualities in morphology, membrane components, hydrolase contents, luminal pH, and distinct degradative capacity, represent the different stages of maturation within the endocytic and autophagic pathways (Saftig and Klumperman, 2009). Mature lysosomes are thus defined as (1) single membrane-bound organelles containing active forms of degradative hydrolases with most having an acidic pH of 4.5–5 optimum; (2) sites of substrate hydrolysis occurring within limiting membranes enriched in glycosylated lysosome-associated membrane proteins 1 and 2 (LAMP1/2); and (3) lacking endosome-specific proteins such as mannose-6-phosphate receptors (Lie and Nixon, 2019; Luzio et al., 2007; Saftig, 2005).

## Axonal lysosome maturation and distribution

The unique compartmental features of neurons add yet another layer of complexity to lysosome maturation. As in other cell types, neuronal lysosomes receive cargos through the endocytic, autophagic, and biosynthetic pathways. Because of their extreme morphological features, neurons require coordinated bidirectional transport mechanisms to maintain a steady-state distribution of the endolysosomal system in the axon, dendrites, and cell body. Degradative lysosomes are predominantly located in the cell body (Cai et al., 2010; Gowrishankar et al., 2017; Gowrishankar et al., 2015; Yap et al., 2018), representing a unique distribution pattern thought to facilitate coordination between recycling and biogenesis pathways in the soma (Lie et al., 2021; Maday et al., 2014). However, degradative lysosomes and lysosome-like organelles have also been found in axons (Farfel-Becker et al., 2019; Farias et al., 2017; Jin et al., 2018; Lee et al., 2011; Roney et al., 2021; Terni and Llobet, 2021) and dendrites (Goo et al., 2017; Padamsey et al., 2017; van Bommel et al., 2019), reflecting the existence of lysosome subpopulations with compartment-specific functions and/or lysosomal organelles at varying degrees of maturation.

Early work measuring the pH of axonal endocytic cargos demonstrated an increasing frequency of acidic organelles as cargos moved from distal to proximal (Overly and Hollenbeck, 1996; Overly et al., 1995), suggesting a retrograde trafficking route of axonal endolysosome maturation. This was speculated to result from an increased likelihood of endosomes to fuse with Golgi-derived vesicles in proximal regions or an increased probability of proton pump activation in the vicinity of the soma (Overly et al., 1995). Additional lines of evidence in support of this model were later reported. Endocytic substrates transited through EEs and LEs along a retrograde transport route toward the cell body (Fig. 1 B; Deinhardt et al., 2006). Similar observations were recently extended to dendrites, further revealing a distinct gradient of degradative activity in neurons. Whereas EEs and LEs were detected throughout the length of dendrites, mature lysosomes positive for LAMP1 and cathepsins B and D (CTSB/D) were largely confined to the cell body and proximal dendritic regions (Yap et al., 2018). Consistently, the Rab7 effector WDR91 involved in early-to-late endosome conversion is required for endosome maturation in neurites, which is essential for dendrite growth and neuronal lysosome function (Liu et al., 2017; Xing et al., 2021). Similarly, impaired retrograde transport of endolysosomal organelles in axons caused an accumulation of immature lysosomes in neurons, leading to decreased proteolytic capability (Cai et al., 2010; Gowrishankar et al., 2017; Gowrishankar et al., 2015; Lee et al., 2011).

While it is well established that enzymatically active degradative lysosomes are enriched in the cell body, their trafficking to and positioning at distal neurites have recently been observed. Acidic organelles positive for pH-based LysoTracker were detected in dendrites and dendritic spines (Goo et al., 2017; Padamsey et al., 2017; van Bommel et al., 2019). LysoTracker signal in dendritic lysosomes was sensitive to treatment with glycyl-L-phenylalanine 2-naphthylamide (Goo et al., 2017; Padamsey et al., 2017), a cell-permeable substrate that induces osmotic lysis following cleavage by the lysosomal hydrolase CTSC (Berg et al., 1994). These studies suggest a population of lysosomes with some functionality located in dendrites and dendritic spines. Consistently, inhibiting lysosomal proteolytic function altered lysosome movement and reduced spine density, thus linking lysosomal degradation capacity to their trafficking and function in dendrites (Goo et al., 2017). Lysosomes were also observed to be recruited to the base of dendritic spines upon local glutamate uncaging (Goo et al., 2017) and to release CTSB through lysosomal exocytosis to regulate dendritic spine plasticity and morphology (Padamsey et al., 2017), supporting a role for lysosomes in dendritic branching and spine density to maintain synaptic homeostasis.

At axon terminals, a study in intact *Drosophila* brains revealed distinct molecular machineries involved in cargo-specific sorting and degradation (Jin et al., 2018). While cargos carrying plasma membrane proteins were transported toward the soma for maturation and clearance, synaptic vesicle proteins were sorted for local degradation executed by cathepsin-L-like protease 1, indicating the presence of lysosome subpopulations in axonal terminals with selective degradation capacity (Jin et al., 2018). Degradative lysosomes have also been detected in axons of primary mouse neurons using various endolysosomal markers and activity-based fluorescent probes specific to active forms of lysosomal hydrolases (Farfel-Becker et al., 2019; Farias et al., 2017; Lee et al., 2011). A recent study provided live-imaging evidence showing dynamic axonal delivery of degradative lysosomes by combining two approaches: (1) applying a set of four activity-based probes for labeling active forms of CTSB/D/L and glucocerebrosidase (GCase); and (2) implementing microfluidic devices to physically and fluidically isolate axons from cell bodies/dendrites, which allows selective labeling of degradative lysosomes in the soma chamber and monitoring their influx into distal axons. Soma-derived degradative lysosomes rapidly transport into distal axons and target autophagosomes and α-synuclein cargos for local degradation (Fig. 1 B; Farfel-Becker et al., 2019). Disrupting axon-targeted delivery of degradative lysosomes or interfering with their proteolytic capacity locally within the axonal chamber induces axonal autophagic stress. Thus, axonal degradation capacity is partially maintained by delivery of “fresh” degradative lysosomes from the lysosomal reservoir in the soma. In addition, a recent study demonstrated that TGN-derived transport carriers (TCs) deliver lysosomal hydrolases to axonal organelles, thus contributing to distal endolysosomal biogenesis and maturation (Fig. 1 B; Lie et al., 2021). Together, these studies further support the notion that while mature lysosomes are predominantly located in the cell body, axons are also degradative compartments, and axonal lysosomes play a critical role in maintaining axonostasis (Farfel-Becker et al., 2019; Farias et al., 2017; Lee et al., 2011).

## Detection of degradative lysosomes in nervous systems

Given emerging lines of evidence implicating lysosome dysfunction and transport defects in the pathogenesis of neurodegenerative diseases (Gowrishankar et al., 2015; Lee et al., 2011; Roney et al., 2021; Xie et al., 2015; Zigdon et al., 2017), specific labeling of degradative lysosomes in nervous systems is critical for advancing knowledge into how lysosome trafficking, distribution, and functionality contribute to neuronal health and disease progression. LAMP1/2 and LysoTracker probes are routinely used as lysosome markers, and LAMP1/2-positive organelles are often referred to as lysosomes in the literature. However, LAMP1/2 are not static components of the lysosomal membrane. Instead, newly synthesized LAMP1/2 exit the TGN through carrier vesicles and enter the endolysosomal pathway (Fig. 1 A), where they are in dynamic equilibrium between endosomes, lysosomes, amphisomes, and autolysosomes (Cook et al., 2004; Deng and Storrie, 1988; Eskelinen et al., 2003; Patterson and Lippincott-Schwartz, 2002; Saftig and Klumperman, 2009). Notably, recent studies in neurons further demonstrated that LAMP1 is distributed among a diverse population of degradative and nondegradative organelles, some of which represent intermediates of different maturation stages within the endocytic, autophagic, and biosynthetic pathways (Cheng et al., 2018; Cheng et al., 2015; Goo et al., 2017; Gowrishankar et al., 2017; Gowrishankar et al., 2015; Lee et al., 2011; Maday et al., 2012; Overly and Hollenbeck, 1996; Overly et al., 1995). These organelles and their intermediates are also acidic to varying degrees (Saftig and Klumperman, 2009), emphasizing the importance of using a combination of endogenous lysosomal markers and activity-based probes to study neuronal lysosomes.

In line with this notion, many LAMP1-labeled organelles in medial/distal dendrites lacked CTSB/D (Yap et al., 2018), and lysosomes labeled with LAMP1 displayed reduced acidity at axon terminals compared with their acidity in dendrites and proximal axons (Farias et al., 2017). In addition, a large portion of overexpressed LAMP1-labeled structures in axons were observed to cotransport with Golgi-derived cargos, suggesting their nondegradative identity (Lie et al., 2021). By applying immuno-EM and high-resolution Airyscan microscopy, a recent study provided a comprehensive and quantitative analysis of LAMP1 distribution in various autophagic and endolysosomal organelles in neurons and showed that a significant portion of LAMP1-labeled organelles lack multiple lysosomal hydrolases (Cheng et al., 2018). Consistently, analysis of the hippocampal CA1 region of the WT mouse brain and cultured neurons revealed that LAMP1-labeled organelles within the neuropil have a lower content of multiple luminal proteases compared with somatic LAMP1-labeled lysosomes (Gowrishankar et al., 2015). Thus, the heterogeneous nature of LAMP1-labeled organelles indicates that neuronal LAMP1 intensity, trafficking, and distribution do not necessarily represent degradative lysosomes under physiological and pathological conditions.

**Figure 2.**
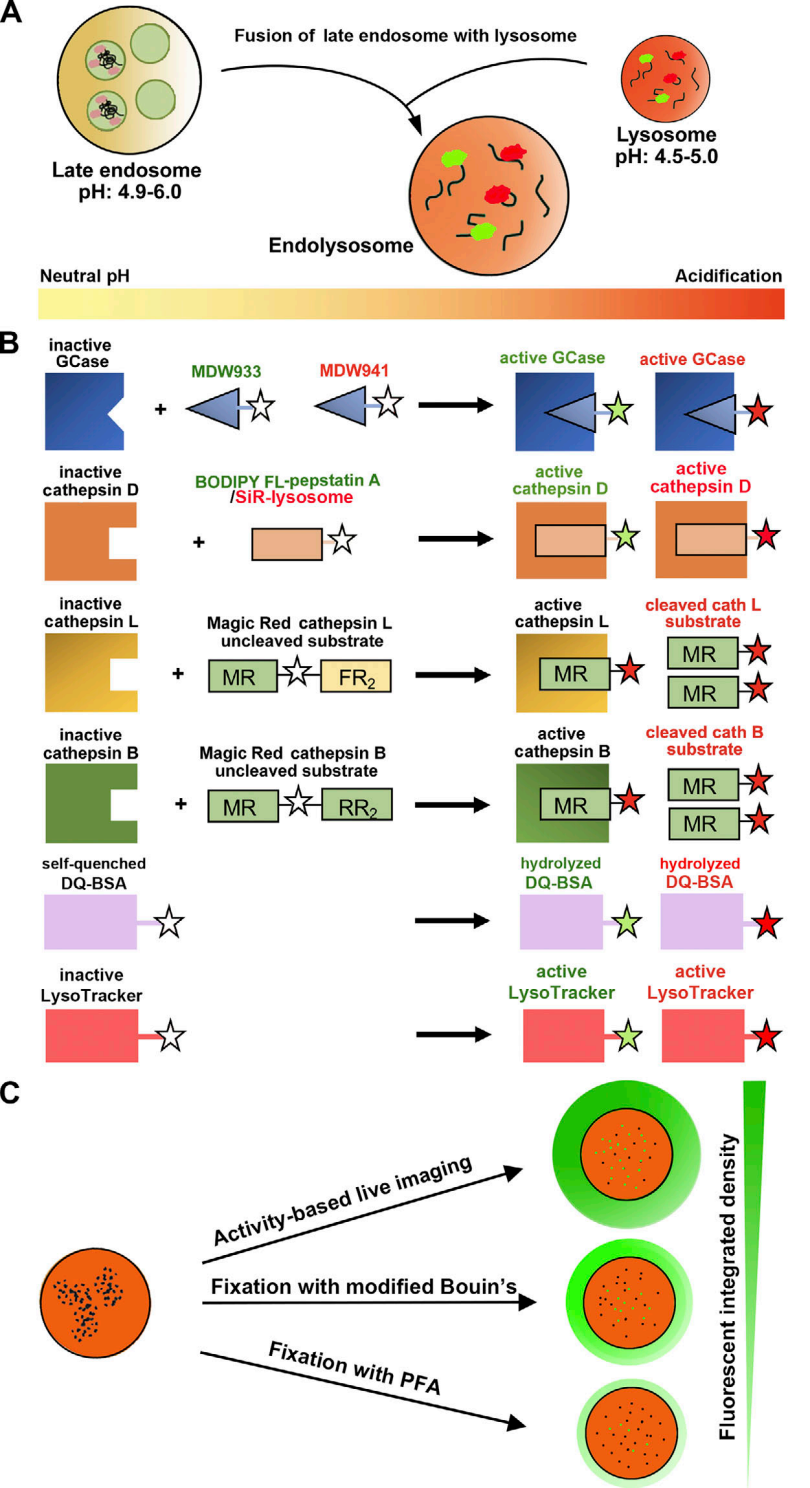
**Schematic illustration of activity-based lysosome probes. (A)** Fluorescent detection of hydrolase activity and degradative capacity using activity-based lysosome probes. These probes enter lysosomes through the endocytic pathway or membrane diffusion, resulting in visualization of punctate fluorescent signals representing lysosomes with degradative activity under acidic conditions. **(B)** Schematic diagram showing lysosome labeling using activity-based lysosome probes. In nonacidic luminal pH environments (left side), lysosomal hydrolases are not in the correct conformation for probe binding or not active for hydrolytic detection. However, in acidic luminal pH environments (right side), lysosomal hydrolases, including GCase and cathepsins D, L, and B, are active and in the correct conformation for probe binding and proteolytic cleavage, resulting in vesicular fluorescent green or red signals (Farfel-Becker et al., 2019). MDW933 (green) and MDW941 (red) are fluorescence-tagged probes that bind the active site of GCase (Witte et al., 2010). BODIPY FL-pepstatin A (green) and silicon rhodamine (SiR)–lysosome (red shown here) are fluorescence-tagged pepstatin A probes that bind the active site of CTSD (Chen et al., 2000; Lukinavičius et al., 2016). Magic Red (MR) substrates consist of a fluorophore bound to a peptide motif (i.e., FR_2_/RR_2_) that targets the molecule for cleavage by specific cathepsins (Creasy et al., 2007). Before cleavage, Magic Red substrates are cell permeable and nonfluorescent. After cleavage, they are membrane impermeable and fluoresce red. DQ-BSA substrates (green or red) are BSA derivatives that are labeled to such a high degree with BODIPY dyes that they are self-quenched but become fluorescent upon proteolytic cleavage of the BSA conjugates (Vazquez and Colombo, 2009). LysoTracker probes (green or red shown here) are cell-permeable dyes that consist of a fluorophore linked to a weak base that is protonated at acidic pH and becomes trapped and thus accumulates in acidic compartments. **(C)** Relative signal detection of lysosomal luminal proteins by live imaging with activity-based probes and by immunostaining after fixation with modified 50% Bouin’s or PFA. Note that lysosomal hydrolases are more sensitively detected by live imaging with activity-based probes or optimally detected by immunostaining after fixation with modified 50% Bouin’s solution versus fixation with PFA.

Together, these studies help clarify criteria that may be used to characterize neuronal lysosomes, which can be defined as acidic LAMP1-positive organelles containing active lysosomal hydrolases and degraded substrates (Fig. 2 A). Therefore, fluorescent detection of hydrolase activity and degradative capacity using activity-based lysosome probes is highly recommended. These activity-based probes are delivered to lysosomes through the endocytic pathway or membrane diffusion, enabling fluorescent detection of hydrolase activity upon fusion events between LEs and lysosomes (Fig. 2 A; Bright et al., 2016). In nonacidic luminal pH environments, lysosomal hydrolases, such as GCase and CTSB/D/L, are not in the correct conformation for probe binding or not active for hydrolytic detection requiring acidic conditions. However, in acidic luminal pH environments, lysosomal hydrolases are active and in the correct conformation for probe binding and proteolytic cleavage, resulting in vesicular fluorescent green or red signals (Fig. 2 B).

A recent study reported that transgenic mice expressing human CTSD (hCTSD) in neurons showed enriched hCTSD puncta in somatodendritic regions of layer 5 cortical neurons and hippocampal CA1 neurons (Lie et al., 2021). Although these hCTSD signals were not readily detected in axonal fibers of the corpus callosum, striatum, and cerebellum in vivo, this study demonstrated that lysosome-like organelles containing endogenous active CTSB and CTSD transport along live neuronal axons in culture (Lie et al., 2021), thus excluding their nature as TGN-derived TCs, in which case hydrolases would not be activated in nonacidic luminal pH environments. This study supports the notion that degradative lysosomes are also distributed in axons. The apparent contradiction between the in vivo and in vitro observations of this study may arise from biological differences between these two systems or technical limitations in detecting in vivo lysosomal luminal hydrolases with varying pH optima that may be more sensitively monitored by live imaging or optimally detected following fixation with Bouin’s solution instead of the routine 4% PFA. Bouin’s fixation was reported to reliably detect lysosomal luminal proteins by immunofluorescence (Harrison et al., 2009; Lin and Lobel, 2001; Zhang et al., 2003). A comparison of these two fixation protocols revealed that a modified 50% Bouin’s fixation condition significantly enhances signal detection of CTSD by almost threefold in cortical neurons (Fig. 2 C), where LAMP1-labeled organelles containing CTSD were 28.6% in dendrites and 30.31% in axons (Cheng et al., 2018). These observations were further confirmed in vivo by coimmunostaining of LAMP1 and CTSD in adult mice after perfusion with 50% Bouin’s solution; LAMP1 signals that colocalized with CTSD were readily detected along dorsal root ganglion axonal bundles (Cheng et al., 2018). Thus, it is optimal to use multiple activity-based lysosome probes combined with modified fixation conditions to detect active lysosomal luminal hydrolases, which are relatively low in abundance within lysosomes positioned along axons and terminals compared with the soma. These combined approaches will help reveal new insights into how degradative lysosomal trafficking, distribution, and functionality support neuronal health and how axonal lysosomes respond to disease conditions in both in vitro and in vivo nervous systems.

## Endolysosome transport machinery

Neurons require efficient transport mechanisms to maintain a steady-state distribution of endolysosomes throughout the cell. Kinesin and dynein motors drive long-distance transport along microtubule (MT) tracks in axons and dendrites, whereas myosin motors mediate short-range movement along actin filaments enriched in growth cones and synaptic regions (Hirokawa et al., 2010). Bidirectional transport in axons and dendrites depends in part on specific motor–adaptor interactions as well as the organization of the MTs within these compartments. In axons, MTs are uniformly polarized, with plus ends oriented outward and minus ends directed inward (Baas et al., 1988). Therefore, kinesin motors mediate anterograde transport toward axon terminals, whereas dynein motors mediate retrograde transport from distal axons toward the cell soma. However, because dendritic MTs exhibit mixed polarity, kinesin and dynein motors may drive lysosome movement in either direction in dendrites. Endolysosomes couple to motor proteins through interactions with adaptors that recruit and assemble transport complexes on their membranes. These adaptors, including small GTPases, effector proteins, and phospholipids, regulate cargo-selective and/or compartment-specific transport.

### Anterograde transport

**Figure 3.**
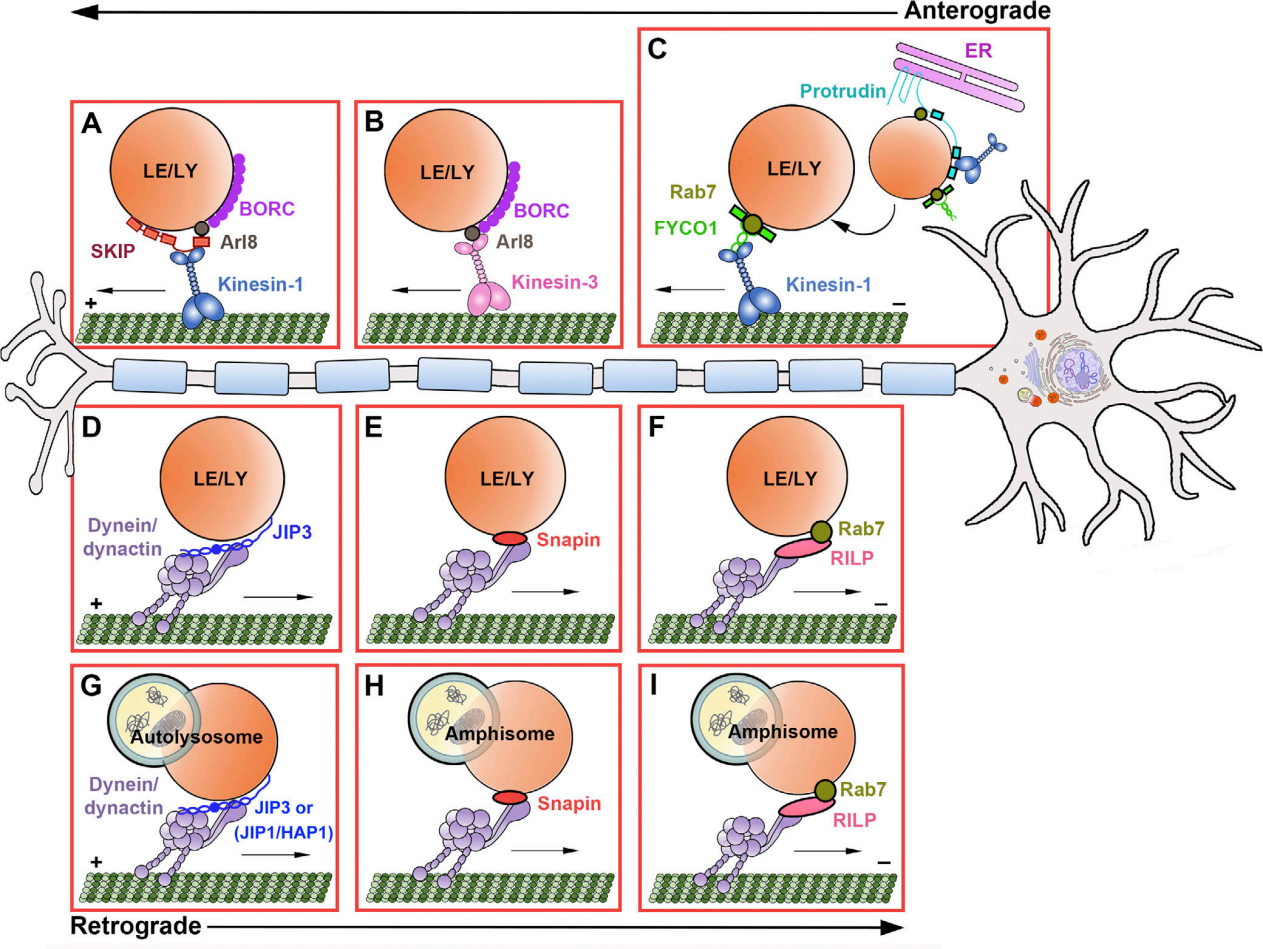
**Axonal endolysosome transport machinery.** Schematic diagram showing motor–adaptor complexes mediating kinesin and dynein motor recruitment to LE/LY membranes for MT-based transport along axons. Note that kinesin motors transport LE/LY toward axon terminals, whereas dynein motors drive axonal organelle movement toward the cell body. **(A)** The BORC–Arl8–SKIP–kinesin-1 complex drives LE/LY transport into axons. BORC associates with lysosomal membranes and recruits Arl8 from the cytoplasm. SKIP then interacts with Arl8 and kinesin light chain, linking LE/LY to kinesin-1 motors. **(B)** Arl8A mediates organelle coupling to kinesin-3 for LE/LY transport into axons. **(C)** The Rab7–FYCO1–kinesin-1 complex mediates anterograde LE/LY transport toward axon terminals. The ER protein protrudin forms contacts with LEs through interaction with Rab7 and phosphatidylinositol 3-phosphate on membranes. Protrudin then transfers kinesin-1 onto organelles through FYCO1, facilitating LE/LY transport toward MT plus ends. **(D)** JIP3 mediates lysosome coupling to dynein-dynactin for retrograde axonal LE/LY transport. **(E)** Snapin mediates LE/LY coupling to dynein-dynactin through DIC interaction for retrograde transport from axon terminals toward the soma. **(F)** The Rab7–RILP–dynein–dynactin complex facilitates retrograde axonal LE/LY movement. RILP links LEs to dynein-dynactin through binding Rab7 and dynactin subunit p150^Glued^. **(G)** JIP3 promotes the retrograde transport of mature autolysosomes in mid-axon and proximal axon regions through dynein scaffolding regulation. Note that other dynein effectors regulate AV motility depending on their maturation and location within axons, including JIP1 in distal axons and HAP1 in mid-axons. **(H)** LE-loaded dynein–snapin complexes are shared by autophagosomes upon their fusion to form amphisomes and drive their retrograde transport toward the soma. **(I)** The Rab7–RILP–dynein–dynactin complex mediates retrograde axonal transport of amphisomes through RILP interaction with Rab7.

The kinesin superfamily is composed of ≥45 genes grouped into 15 families, designated kinesin-1 to kinesin-14B (Lawrence et al., 2004; Miki et al., 2001). In general, kinesin proteins contain a motor domain that attaches to MTs and a tail domain that interacts directly with cargos or their adaptors to mediate cargo loading (Hirokawa and Noda, 2008). Most kinesin superfamily proteins (KIFs) transport organelles toward MT plus ends to facilitate anterograde movement. Mechanisms driving anterograde lysosome transport identified in nonneuronal cells have recently been investigated in neurons. The BORC–Arl8–SKIP–kinesin-1 complex drives lysosome transport into distal axons but not dendrites (Fig. 3 A; Farias et al., 2017). BLOC-one related complex (BORC) is a multiprotein complex that associates with the lysosome surface and is required for recruitment of the small GTPase Arl8 from the cytoplasm (Pu et al., 2015). Active Arl8 then recruits and activates its effector SifA and kinesin-interacting protein (SKIP) from an autoinhibited state (Keren-Kaplan and Bonifacino, 2021), linking lysosomes to kinesin-1 motors via kinesin light chain (Rosa-Ferreira and Munro, 2011). Interference with multiple components of this complex selectively impairs axonal lysosome availability, leading to altered axonal homeostasis (Farfel-Becker et al., 2019; Farias et al., 2017). Mice with spontaneous mutations in *Borcs7* (encoding a BORC subunit) develop progressive axonal dystrophy and motor dysfunction, indicating a critical role of BORC-dependent axonal lysosome transport for maintaining axon integrity and neuron function in vivo (Snouwaert et al., 2018).

Members of the kinesin-3 family also mediate anterograde lysosome transport in neurons. The kinesin-3 family member KIF1A, previously observed to couple lysosomes to distinct MT tracks in nonneuronal cells (Guardia et al., 2016), was recently shown to drive lysosome transport to the periphery of neurons via Arl8A (Fig. 3 B; Hummel and Hoogenraad, 2021). Interestingly, Arl8 interaction with KIF1A or its ortholog UNC-104 also mediates the plus end–directed transport of dense core vesicles (DCVs; Hummel and Hoogenraad, 2021; Lund et al., 2021) and synaptic vesicle precursors (SVPs; Niwa et al., 2017; Vukoja et al., 2018; Wu et al., 2013). Overlap between the axonal transport machinery of DCVs and lysosomes was also observed in *Drosophila* brains, where Arl8 promoted DCV movement into axons while Rab2 was required for the bidirectional transport of both DCVs and lysosomal organelles along axonal processes (Lund et al., 2021). Interestingly, LEs/lysosomes can also act as “hitchhiking” platforms for RNA granule transport (Cioni et al., 2019; Corradi et al., 2020; Liao et al., 2019). These studies suggest that lysosomes and other cargos may share common transport machinery for long-distance movement in axons. However, while BORC was observed to mediate the anterograde transport of SVPs in axons of *Caenorhabditis elegans* neurons (Niwa et al., 2017; Zheng et al., 2014), a subsequent study demonstrated that BORC was dispensable for the axonal transport of SVPs in mammalian neurons (De Pace et al., 2020), an observation that may suggest species-specific differences in coupling kinesin motors to these cargos.

Kinesin-1 coupling to LEs and lysosomes can also be achieved across membrane contact sites with the ER. For example, the ER protein protrudin forms contacts with LEs through interactions with Rab7 and phosphatidylinositol 3-phosphate on endolysosomal membranes. This allows protrudin-mediated transfer of kinesin-1 onto LEs through the motor adaptor FYCO1, facilitating endolysosome transport toward MT plus ends to promote protrusion and neurite outgrowth (Fig. 3 C; Matsuzaki et al., 2011; Palomo-Guerrero et al., 2019; Raiborg et al., 2015). In addition, contact sites between ER tubules and endolysosomes at pre-axonal regions can drive lysosome transport into axons (Ozkan et al., 2021). These ER–lysosome contacts regulate lysosome size through kinesin-1–mediated fission, resulting in the subsequent delivery of newly reformed lysosomes to distal axons. Indeed, KIF5B clustering on phosphatidylinositol 4,5-biphosphate–enriched membrane microdomains drives autolysosomal tubulation in nonneuronal cells (Du et al., 2016), thus linking motor-driven tubulation to lysosome maturation and transport.

### Retrograde transport

Cytoplasmic dynein is the major motor protein that drives endolysosome transport toward MT minus ends. It is composed of two heavy chains with ATPase activity, two intermediate chains, two light intermediate chains, and several light chains (Reck-Peterson et al., 2018). In addition, the associated protein complex dynactin interacts with dynein and is required for dynein association with endolysosomes (Burkhardt et al., 1997). Endolysosome coupling to dynein motors is mediated by several adaptor protein complexes recruited under various conditions. Dynein loading onto endolysosome membranes is mediated by JNK-interacting protein 3 (JIP3; Fig. 3 D). JIP3 and its homologs, Sunday Driver (SYD) in *Drosophila* and UNC-16 in *C. elegans*, associate with lysosomes (Drerup and Nechiporuk, 2013; Gowrishankar et al., 2017) and interact with dynein light intermediate chain (Arimoto et al., 2011) and dynactin subunit p150^Glued^ (Cavalli et al., 2005). In zebrafish, Jip3 was observed to colocalize with lysosomes moving bidirectionally along axons; loss of Jip3 reduced lysosome association with dynein motors, and loss-of-function *jip3* mutants displayed lysosome accumulations in axonal terminal swellings, consistent with defects observed in retrograde lysosome movement (Drerup and Nechiporuk, 2013). This was further supported by the observation that *Jip3* knockout (KO) mouse dystrophic axons contain immature lysosomes with low levels of proteases, representing a population of lysosomal intermediates distinct from mature lysosomes in the cell body (Gowrishankar et al., 2017). Interestingly, these axonal organelle accumulations were also observed in *JIP3* KO human induced pluripotent stem cell–derived neurons, which were accompanied by disruptions in the axonal cytoskeleton and worsened by KO of homologous *JIP4* (Gowrishankar et al., 2021; Rafiq et al., 2022). JIP4 was previously characterized in nonneuronal cells as a dynein adaptor recruited to lysosomes through the lysosomal integral membrane protein TMEM55B (Willett et al., 2017). However, because JIP3/SYD/UNC-16 also interacts with components of the kinesin-1 motor to mediate anterograde cargo transport (Arimoto et al., 2011; Bowman et al., 2000; Byrd et al., 2001; Huang et al., 2011; Sun et al., 2017; Verhey et al., 2001), it was proposed that JIP3 and its homologs may serve as scaffolding proteins regulating bidirectional transport, highlighting how anterograde and retrograde transport complexes may be integrated to coordinate lysosomal motility and distribution throughout the axon (Fu and Holzbaur, 2014).

Snapin acts as an adaptor protein that links dynein motors to LEs through interactions with dynein intermediate chain (DIC) and thus facilitates retrograde LE transport from axonal terminals toward the soma (Fig. 3 E; Cai et al., 2010). Deleting *Snapin* reduces dynein association with LEs in neurons, whereas overexpressing snapin enhances motor-driven tubular endolysosome formation in the soma. *Snapin* KO neurons exhibit phenotypes linked to impairments in retrograde LE transport and their maturation into lysosomes, including aberrant accumulation of immature lysosomes, reduced lysosomal proteolysis, and impaired autolysosome clearance. Consistent with its role in late endocytic trafficking and maturation in axonal terminals, snapin promotes the sorting of synaptic vesicle proteins to the endolysosomal pathway for degradation (Di Giovanni and Sheng, 2015) and enhances endosome-to-Golgi retrieval of cation-independent mannose-6-phosphate receptors to support neuronal lysosome biogenesis (Tammineni et al., 2017a). Interestingly, snapin is also copurified with the BORC complex (Pu et al., 2015) that is involved in kinesin-driven axonal lysosome movement (Farias et al., 2017), although its direct role in axonal anterograde lysosomal transport remains to be examined.

An additional mechanism for dynein recruitment to LEs/lysosomes involves Rab7 and its effector Rab7-interacting lysosomal protein (RILP; Cantalupo et al., 2001; Jordens et al., 2001), which links endolysosomes to dynein-dynactin through interaction with Rab7 and the dynactin subunit p150^Glued^ (Johansson et al., 2007). Interestingly, in nonneuronal cells, the Rab7–RILP–dynein–dynactin complex associates with ORP1L, which regulates LE association with dynein-dynactin in response to endosomal cholesterol levels through contacts with the ER (Johansson et al., 2007). In neuronal cells, RILP regulates dynein attachment to axonal LEs through interaction with Rab7; RILP knockdown in rat cortical neurons significantly decreased the retrograde transport of LEs and increased their stationary population, consistent with a role for RILP in driving retrograde LE movement along axons (Fig. 3 F; Khobrekar et al., 2020).

## Regulation of neuronal lysosome trafficking and positioning

Lysosome trafficking and positioning throughout axons and dendrites change in response to the cellular environment and neuronal activity. This enables lysosomes to respond dynamically to meet various homeostatic demands and adapt to specific stimuli, including synaptic inputs. For example, lysosomes traffic to dendritic spines in an activity-dependent manner; synaptic activation increased the number of lysosome-containing dendritic spines, and local glutamate uncaging at an individual spine increased lysosome positioning at its base (Goo et al., 2017). Interestingly, disruption of actin filaments (F-actin) by latrunculin A increased dendritic lysosome motility, suggesting a role for F-actin in positioning lysosomes in spines and synapses in response to neuronal activity (Goo et al., 2017; van Bommel et al., 2019). These synaptic lysosomes may contribute to dendritic AV degradation, given that synaptic stimulation was also observed to reduce dendritic AV motility and increase the percentage of degradative autolysosomes in dendrites (Kulkarni et al., 2021). Further work revealed that dendritic actin patches act as both a physical barrier and an anchoring platform for lysosome stalling near synapses via the actin-based motor myosin Va (van Bommel et al., 2019). Together, these findings demonstrate an actin-mediated mechanism for lysosome recruitment to dendritic spines in response to synaptic activity, when postsynaptic membrane protein recycling or turnover may be in high demand.

Neuronal lysosomes also traffic to and fuse with the plasma membrane in an activity-dependent manner. In dendrites, lysosomal calcium release evoked by back-propagating action potentials triggered exocytosis of CTSB into the extracellular space, which activated metalloproteinase 9 signaling to regulate dendritic spine growth and plasticity (Padamsey et al., 2017). Consistently, inhibiting lysosomal calcium signaling or CTSB release prevented such activity-dependent spine remodeling. Similarly, at axonal terminals, activity-dependent exocytosis of synaptic organizer protein Cbln1 and CTSB from lysosomes supported synapse modification (Ibata et al., 2019). These findings further support the role of synaptic lysosomes in the activity-dependent regulation of synaptic homeostasis.

Moreover, lysosomes redistribute following environmental and metabolic changes. For instance, LE/lysosome (LY) transport and positioning responds to alterations in cytoplasmic pH. Acidification of the cytosol causes lysosome dispersal into neuronal processes, whereas cytosolic alkalization shifts LE/LY distribution to the cell body (Heuser, 1989; Parton et al., 1991), suggesting that motor protein complexes regulate LE/LY motility by sensing intracellular pH. In addition, lysosomes have emerged as signaling organelles that sense alterations in the metabolic environment and coordinate a response in lysosome transport and positioning. For example, the enzyme carnitine palmitoyltransferase 1C regulates LE/LY abundance at axon terminals depending on the nutrient status of the cell (Palomo-Guerrero et al., 2019). Under sufficient nutrient conditions, sensing of the fatty acid precursor malonyl CoA by carnitine palmitoyltransferase 1C enhanced protrudin transfer of kinesin-1 to LEs through FYCO1, thereby promoting lysosome redistribution to the cell periphery to support axon growth. However, under metabolic stress, this process was largely prevented. Thus, regulation of lysosome transport and distribution by sensing nutrient availability allows coordination of neurite outgrowth during conditions favorable to biosynthesis. Together, these regulated transport mechanisms allow neurons to adapt to changing cellular conditions by effectively trafficking and positioning lysosomes throughout the cell.

## Bidirectional endolysosome transport facilitates axonal autophagic clearance

Degradation of autophagic cargos depends on their dynamic interactions with endolysosomes, which supply the hydrolytic enzymes needed for substrate degradation (Luzio et al., 2007). Both the anterograde and retrograde transport of lysosomes are required to mediate encounters with autophagosomes for cargo degradation (Jia et al., 2017; Korolchuk et al., 2011). This process is particularly challenging in neurons due to their highly polarized morphology with extremely long axons. While enzymatically active degradative lysosomes are relatively enriched in the cell body (Cai et al., 2010; Gowrishankar et al., 2017; Gowrishankar et al., 2015; Yap et al., 2018), mature lysosomes and lysosome-like organelles are also found in distal axons (Cheng et al., 2018; Farfel-Becker et al., 2019; Farias et al., 2017; Gowrishankar et al., 2017; Jin et al., 2018; Lee et al., 2011). These degradative lysosomes transport bidirectionally along axons to enable dynamic interactions with autophagosomes, thus facilitating autophagic maturation and clearance.

In axons, autophagosomes predominantly form at distal terminals (Maday et al., 2012), then subsequently transport retrogradely toward the cell body for maturation and degradation, a process driven by dynein motors (Katsumata et al., 2010; Kimura et al., 2008; Lee et al., 2011; Maday et al., 2012). Because mature acidic lysosomes are enriched in proximal axonal regions and the soma, the majority of autophagosomes generated in axon terminals undergo (1) fusion with distal LEs to form amphisomes, and then (2) retrograde transport toward the soma where they mature into autolysosomes for degradation (Fig. 1 B). To achieve effective autophagic flux in axons, during their retrograde trafficking route, these axonal autophagosomes encounter and fuse with lysosomes that are anterogradely delivered from the soma to become autolysosomes (Farfel-Becker et al., 2019; Farias et al., 2017), thus acquiring lysosomal hydrolases and an acidified environment, leading to cargo degradation (Lee et al., 2011; Maday et al., 2012). Consistently, the majority of axonal autophagosomes colocalize with LE marker Rab7 (Cheng et al., 2015; Lee et al., 2011; Maday et al., 2012), as well as activity-based lysosome probes (Farfel-Becker et al., 2019).

Thus, the maturation of these distal AVs into autolysosomes is tightly linked to their retrograde transport. Scaffolding proteins, including JIP1, HTT-associated protein 1 (HAP1), and JIP3 (Fig. 3 G), regulate autophagosome motor activity to ensure their retrograde processivity (Fu et al., 2014; Hill et al., 2019; Wong and Holzbaur, 2014), a feature observed in several neuronal systems. Interestingly, as axonal autophagosomes travel along axons, they acquire these multiple effectors that sequentially regulate dynein activity and thus autophagosome motility, depending on their maturation status and location within axons (Cason et al., 2021). Thus, fusion between autophagosomes and endolysosomes not only facilitates cargo maturation but also influences motor protein acquisition and activity. Indeed, the transport of LEs and lysosomes is coordinated with the recruitment of fusion components (Jia et al., 2017; van der Kant et al., 2013). This enables lysosomes to couple transport and fusion with degradation. This may be particularly important in neuronal axons, which span several tens to hundreds of centimeters away from the cell body.

Although dynein-driven retrograde transport of autophagosomes was suggested, a fundamental question remained as to how autophagosomes generated at distal axons acquire dynein motors for retrograde transport toward the soma. Using live rat dorsal root ganglion neurons combined with molecular disruption of autophagosome fusion with LEs or impairment of dynein–snapin (motor–adaptor) coupling, a study revealed a motor-adapter sharing model in which LE-loaded dynein–snapin complexes are shared by autophagosomes upon their fusion (Fig. 3 H; Cheng et al., 2015). Blocking AV-LE fusion reduced dynein recruitment to AVs, thus immobilizing them in distal axons. Consistently, interfering with dynein–snapin coupling impaired AV retrograde transport, resulting in autophagosome accumulation in distal neurites and synaptic terminals. Thus, this motor–adaptor sharing mechanism allows neurons to adapt a more efficient maintenance of axonostasis by removing distal AVs engulfing aggregated proteins and dysfunctional organelles. Consistent with a role for LEs in driving axonal AV motility and maturation, RILP binds both LC3 and Rab7 to mediate dynein recruitment to amphisomes for transport toward the cell body (Fig. 3 I; Khobrekar et al., 2020).

Recent evidence further revealed the role of lysosomes in axonal autophagosome maturation and clearance. Lysosomes that transport from the soma into axons target autophagic cargo to facilitate local AV maturation and cargo degradation. These lysosomes contain active lysosomal enzymes and are continuously delivered to distal axons from the cell body (Farfel-Becker et al., 2019). Disrupting axonal lysosome delivery by interfering with multiple components of the BORC–Arl8–SKIP–kinesin-1 complex results in the accumulation of autophagosomes in distal axons, highlighting a critical role for anterograde lysosome transport in the maintenance of axonal autophagic clearance (Farfel-Becker et al., 2019; Farias et al., 2017). Altogether, efficient clearance of axonal autophagosomes requires coordination between their retrograde transport toward the soma and the anterograde delivery of soma-derived degradative lysosomes to distal axons. This concept is supported by several studies showing that impairments in either transport process contribute to axonal autophagic stress and neurodegeneration (Cheng et al., 2015; Farfel-Becker et al., 2019; Farias et al., 2017; Tammineni et al., 2017b; Wong and Holzbaur, 2014). This bidirectional transport model facilitates efficient degradation and removal of axonal cargos during their opposing trafficking routes (Fig. 1 B).

## Axonostasis failure in neurodegenerative diseases

Lysosome dysfunction and impaired trafficking are linked to the pathogenesis of rare and common neurodegenerative diseases, including frontotemporal lobar degeneration (FTLD), Alzheimer’s disease (AD), amyotrophic lateral sclerosis (ALS), Huntington’s disease (HD), Parkinson’s disease (PD), and lysosomal storage disorders (LSDs) such as Niemann-Pick disease type C (NPC; Boland and Platt, 2015; Menzies et al., 2017; Wallings et al., 2019; Wang et al., 2018). In these diseases, axonal dystrophy is a characteristic pathologic feature. These dystrophic axons contain accumulated organelles related to the endolysosomal–autophagic pathways, reflecting defects in their maturation and/or clearance. Because these organelle trafficking routes are dynamic and span the length of axons, defects at various steps have been described to contribute to the aberrant accumulation of LE- and lysosome-like organelles and AVs within swollen axons of these diseased neurons.

**Figure 4.**
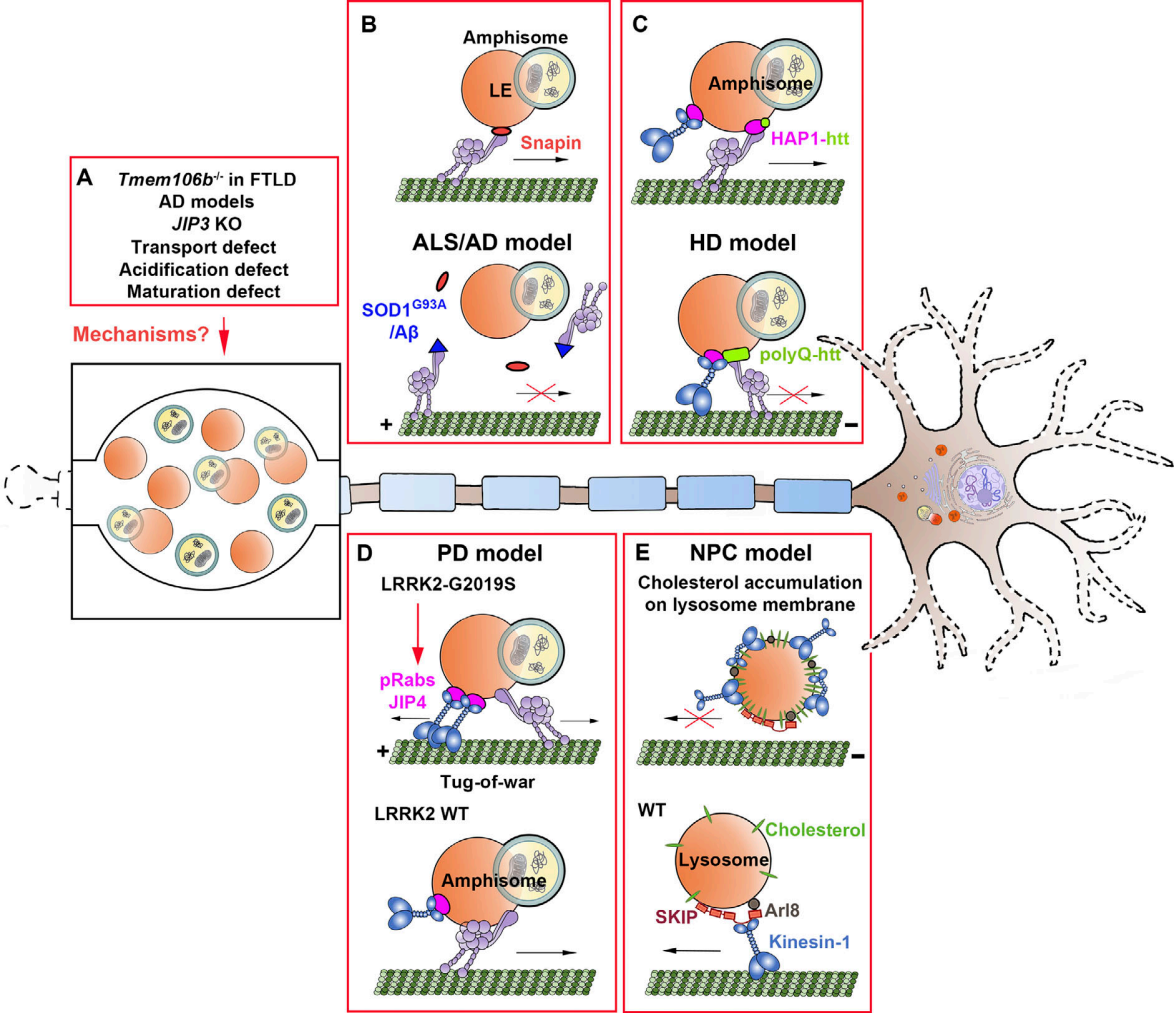
**Axonostasis failure linked to impaired axonal transport and maturation of endolysosomes and autophagosomes in neurodegenerative diseases. (A)** In models linked to FTLD and AD, organelles related to the endolysosomal–autophagic pathways abnormally accumulate within dystrophic axonal swellings. Although these models are associated with defects in lysosome acidification, maturation, and/or trafficking, the specific mechanisms contributing to axonal organelle accumulation in the models remain an active area of investigation. **(B)** In WT neurons, snapin mediates coupling of dynein motors to LEs for driving LE or amphisome retrograde transport toward the soma (top). Mutant SOD1 induced by a familial ALS–linked pathogenic mutation (SOD1^G93A^), as well as AD-linked pathogenic amyloid-β (Aβ) oligomers, disrupt dynein–snapin coupling in axons, thus reducing dynein motor recruitment to LEs or amphisomes and impairing their retrograde transport (bottom). **(C)** In WT neurons, huntingtin (htt) and HAP1 scaffolding proteins regulate dynein motor activity on amphisomes for processive retrograde transport (top). Pathogenic polyglutamine-expanded huntingtin (polyQ-htt) alters its association with HAP1, thus disrupting retrograde amphisome transport and degradation (bottom). **(D)** In neurons with normal LRRK2 activity, axonal amphisomes display processive dynein-driven retrograde transport to facilitate efficient AV maturation through fusion events with LEs and lysosomes in axons (bottom). Hyperactive LRRK2 kinase activity induced by the G2019S pathogenic mutation increases phosphorylation of Rab GTPases (pRabs), which enhances recruitment of the kinesin-motor-adaptor JIP4 to autophagosome membranes, resulting in abnormal kinesin activation. This leads to an unproductive tug-of-war between anterograde and retrograde motors that disrupts retrograde axonal AV processivity and maturation (top). **(E)** In WT neurons, the Arl8–SKIP–kinesin-1 complex assembles on lysosome membranes in the soma to drive anterograde lysosome transport into axons (bottom). In NPC neurons, altered membrane lipid composition on somatic lysosomes sequesters Arl8 and kinesin-1 independently of SKIP, thus impairing lysosome transport to distal axons (top).

FTLD is a clinically and pathologically heterogenous neurologic disorder characterized by degeneration of the frontal and temporal lobes, leading to dementia and progressive behavioral and/or speech abnormalities (Olney et al., 2017). Genetic risk variants in *TMEM106B*, encoding a transmembrane protein primarily localized on LEs and lysosomes, have been linked to several neurodegenerative diseases, including FTLD (Van Deerlin et al., 2010). TMEM106B deficiency results in impaired lysosome acidification, causing a reduction in the activities of multiple hydrolytic enzymes (Klein et al., 2017). Notably, TMEM106B-deficient mice develop axonal swellings at the axon initial segment, leading to motor neuron (MN) dysfunction and progressive behavioral deficits (Luningschror et al., 2020). Axonal swellings of TMEM106B-deficient mice contained large lysosome-like vacuoles that were positive for LAMP1 and negative for LysoTracker and CTSD, representing a population of immature endolysosomal intermediates (Fig. 4 A). TMEM106B deficiency was further observed to result in axonal lysosome transport defects and impaired degradation of lysosomal cargos, suggesting a link between TMEM106B-associated lysosomal deficits and retrograde sorting at the axon initial segment (Luningschror et al., 2020). In addition, promoting a balance between anterograde and retrograde lysosome transport was observed to restore dendrite loss in TMEM106B knockdown neurons, further suggesting a link between lysosome mistrafficking and neurodegeneration (Schwenk et al., 2013).

Notably, mutations in presenilin 1 that cause familial forms of AD disrupt lysosome function and autophagy (Cataldo et al., 2004; Coen et al., 2012; Lee et al., 2015; Lee et al., 2010; Neely et al., 2011; Tong et al., 2021), linking lysosomal impairments directly to AD. Lysosome acidification defects have also been linked to the characteristic axonal dystrophy of AD (Fig. 4 A; Lee et al., 2011). Studies into AD brain pathology in postmortem human samples (Nixon et al., 2005) and mouse models of AD (Gowrishankar et al., 2015) revealed striking accumulations of degradative, lysosome-like organelles within AD dystrophic axons. These aberrantly accumulated organelles were predominantly characterized as AVs, multivesicular bodies, multilamellar vesicles, and autolysosomes (Nixon et al., 2005). Further studies in mouse models of AD revealed that these organelles are positive for LAMP1 and deficient in multiple lysosomal hydrolases, including CTSB/D/L and asparaginyl endopeptidase (Gowrishankar et al., 2015). Interestingly, inhibition of lysosomal proteolytic function by leupeptin or bafilomycin A1 treatment slowed the axonal transport of both autophagosomes and endolysosomes, leading to their selective accumulation within dystrophic axons characteristic of AD (Lee et al., 2011). These results indicate that blocking the retrograde trafficking of degradative organelles contributes to their axonal accumulation and impaired maturation in AD axonal swellings. Similar organelle accumulations occur in *Jip3* KO mouse neurons, which were characterized as lamellar, multivesicular, and electron-dense organelles with lysosome morphology (Gowrishankar et al., 2017). Because these accumulated organelles in *Jip3* KO neurons resemble a population of intermediates derived from endocytic and autophagic pathways that are formed distally, they are thought to result from a block in their retrograde transport, leading to impaired maturation and degradation of these organelles (Fig. 4 A).

Several pathogenic proteins that associate with AD, as well as ALS and HD, disrupt dynein coupling to LEs and/or amphisomes, thus providing mechanistic insight underlying impaired retrograde transport of these organelles under pathological conditions. In an AD-linked mouse model, amyloid-β oligomers associate with dynein motors and competitively disrupt dynein–snapin coupling, leading to impaired dynein motor recruitment to LEs and amphisomes and thus accumulation of axonal AVs (Fig. 4 B; Tammineni et al., 2017b). Similarly, in a familial ALS-linked mouse model with the G93A mutation in human *SOD1*, mutant SOD1 (hSOD1^G93A^) interacts with DIC (Zhang et al., 2007) and interferes with dynein loading onto LEs, resulting in impaired retrograde transport and autophagy-lysosomal deficits in ventral root axons (Fig. 4 B; Xie et al., 2015). Further in vitro and in vivo studies revealed that progressive lysosomal deficits and impaired autophagic clearance are early familial ALS–linked pathological events that disrupt degradation of damaged mitochondria in spinal MN axons. Mitochondria dysfunction is a common pathologic trigger contributing to MN function decline and reduced MN survival in ALS-linked pathogenesis. Early autophagy-lysosomal deficits in hSOD1^G93A^MNs were effectively rescued by introducing a *Snapin* transgene in vitro and in vivo. By competing with hSOD1^G93A^ for binding to DIC, snapin recruits dynein motors to LEs for driving retrograde transport, which rescues lysosome deficits in MNs in vivo, slows MN degeneration, and ameliorates the disease phenotype (Xie et al., 2015). Therefore, enhancing clearance of damaged mitochondria and mutant protein aggregates by up-regulating endolysosomal retrograde trafficking may be a potential therapeutic strategy for ALS and perhaps other neurodegenerative diseases. Consistently, HD-linked pathogenic polyglutamine expansions in huntingtin disrupt retrograde autophagosome transport, leading to a defect in axonal AV maturation and degradation (Fig. 4 C; Wong and Holzbaur, 2014). These studies further highlight the importance of retrograde transport for endolysosomal maturation and the clearance of autophagic organelles from distal axons.

Impaired axonal transport has also been linked to PD. In sporadic cases, axonal spheroid-like structures composed of synuclein aggregates were observed in postmortem patient brains (Galvin et al., 1999; Braak et al., 2003). Changes in kinesin and dynein protein levels were also observed, suggesting an association between α-synuclein aggregation and impaired axonal organelle transport (Chu et al., 2012; Chung et al., 2009). Altered axonal transport has also been linked to inherited forms of PD. For instance, leucine-rich repeat kinase 2 (LRRK2) has been implicated in the autophagy–lysosomal pathway in age-dependent dopaminergic neurodegeneration (Giaime et al., 2017). The most frequent pathogenic hyperactivation mutation in *LRRK2* (G2019S) leads to enhanced recruitment of JIP4 to AV membranes, inducing abnormal activation of the anterograde motor kinesin (Boecker et al., 2021). This results in an unbalanced “tug-of-war” between anterograde and retrograde motors, leading to decreased processivity of AV retrograde transport for maturation (Fig. 4 D). LRRK2 kinase inhibition in these mutant LRRK2 neurons rescued axonal AV transport, further implicating pathogenic LRRK2 kinase activity in disrupted retrograde axonal transport and PD pathogenesis.

In neurodegenerative LSDs such as NPC, axonal dystrophy is also a major pathologic feature. Interestingly, among several LSDs, the organelles that accumulate within dystrophic axons are ultrastructurally distinct from the lysosome inclusions observed in the cell body (Walkley et al., 2010). Instead, these accumulated axonal organelles resemble each other, suggesting that they may result from or converge on a common cellular defect that leads to impairments in axonal transport and the removal of these organelles from axons. In NPC, endocytic and autophagic organelles accumulate within dystrophic axons (Boland and Platt, 2015; Walkley et al., 2010; Walkley and Suzuki, 2004) and predominantly include multivesicular bodies, multilamellar vesicles, and AVs in NPC mice (Roney et al., 2021). The altered lipid composition within NPC lysosome membranes abnormally sequesters the anterograde motor kinesin-1 and motor-adaptor Arl8 independently of SKIP, resulting in reduced lysosome transport from the soma into axons that disrupts maturation of axonal AVs during their retrograde transport route (Roney et al., 2021). Notably, lowering lysosomal lipid levels with 2-hydroxypropyl-β-cyclodextrin rescues axonal lysosome transport (Prabhu et al., 2021) and their delivery into axons, thus reducing axonal autophagic stress and neuron death in NPC (Fig. 4 E; Roney et al., 2021).

## Conclusions and perspectives

Significant progress has been made toward better understanding the endolysosomal system in neurons and highlighted the importance of coordinated bidirectional transport of axonal endolysosomal and autophagic organelles in the maintenance of local lysosomal functionality and axonostasis. Emerging lines of evidence support the notion that failure in this dynamic process disrupts axonostasis, leading to axonal dystrophy and contributing to the pathogenesis of rare and common neurodegenerative diseases. However, many open questions remain. One of the most urgent and critical questions is, why do neurons need multiple motor–adaptor transport complexes to mediate targeted axonal endolysosomal transport? There are several possibilities: these motor adaptors could (1) be differentially regulated in response to certain stimuli and signaling pathways; (2) associate with unique endolysosome subpopulations at different maturation stages or with specific functions; (3) selectively target to organelles within different segments of long axons; (4) play cell type–specific roles depending on their expression levels within a given neuronal subtype; or (5) serve overlapping or partially redundant functions. Therefore, further work is needed to examine these possibilities, which will conceptually advance our understanding of how these motor–adaptor transport complexes are regulated and coordinated to sustain efficient clearance capacity in distal regions under physiological conditions and become disrupted under pathological conditions.

Moreover, many other important issues also need to be addressed in future studies. For example, do neuronal lysosomes in the periphery or distal regions serve as environmental or metabolic sensors to activate transcriptional pathways in the soma and promote lysosomal adaptation to stress? What are the upstream signaling events that regulate neuronal lysosome trafficking within axons and positioning at sites of high axonostatic demand or under autophagic stress? What role do cytoskeletal modifications play in modulating axonal lysosomal movement? How do interactions with other organelles such as the ER or mitochondria contribute to lysosome positioning and local degradation capacity in axons? Do these dynamic events change throughout development and aging? Addressing these questions will help advance our understanding of neuronal endolysosomal transport and lysosomal functionality under physiological conditions and how impaired trafficking and lysosomal dysfunction contribute to neurodegenerative diseases. Understanding the disease-linked mechanisms underlying axonostatic failure is critical for designing potential therapeutic strategies that target axonostatic restoration to support neuronal survival and function.
